# Supplementary material for: The link between liver fat and cardiometabolic diseases is highlighted by genome-wide association study of MRI-derived measures of body composition
Source: Commun Biol. 2022 Nov 19;5:1271. doi: 10.1038/s42003-022-04237-4 (PMC9675774; doi:10.1038/s42003-022-04237-4)
Supplement: Supplementary file 2 — Supplementary Information-New [file 42003_2022_4237_MOESM2_ESM.pdf]

Supplementary Information

Visceral adipose tissue

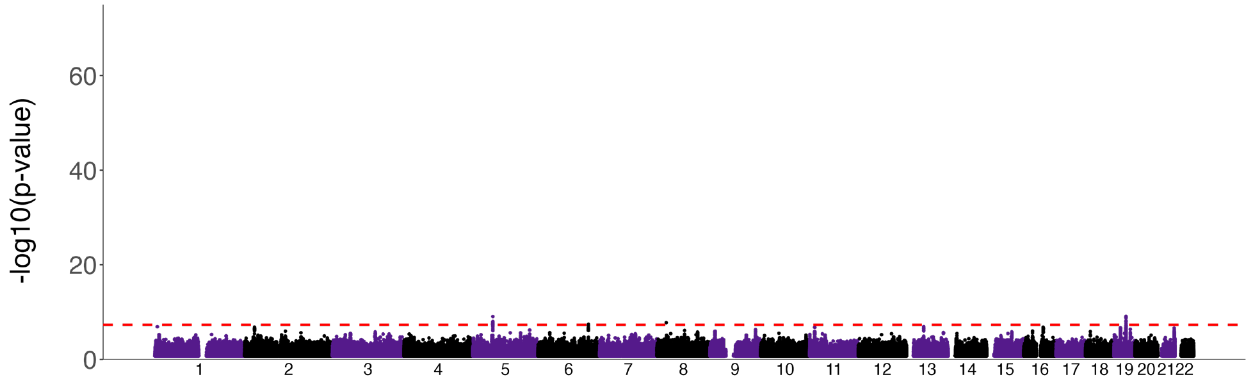

Abdominal subcutaneous adipose tissue

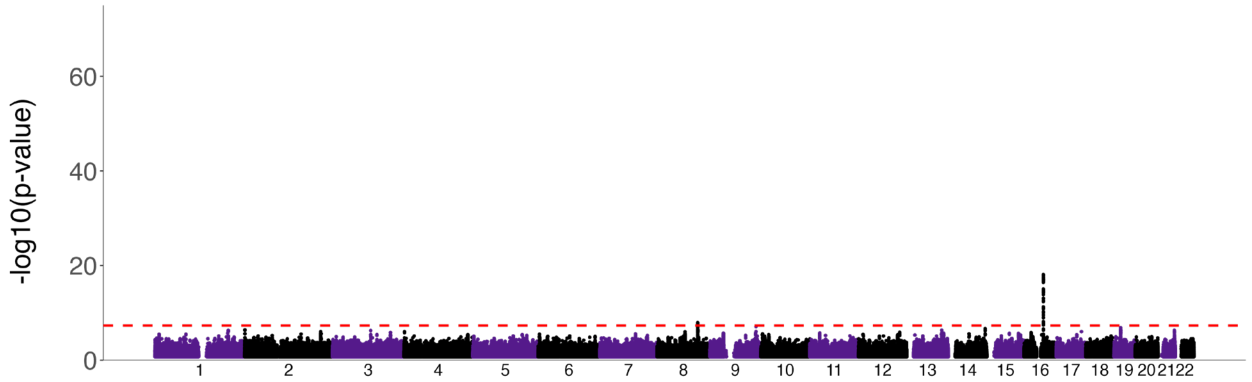

Anterior thigh muscle volume

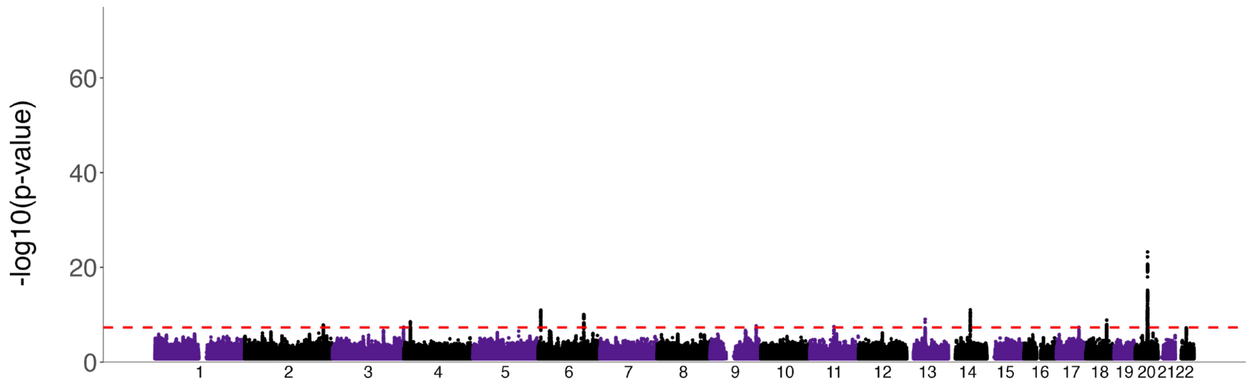

Posterior thigh muscle volume

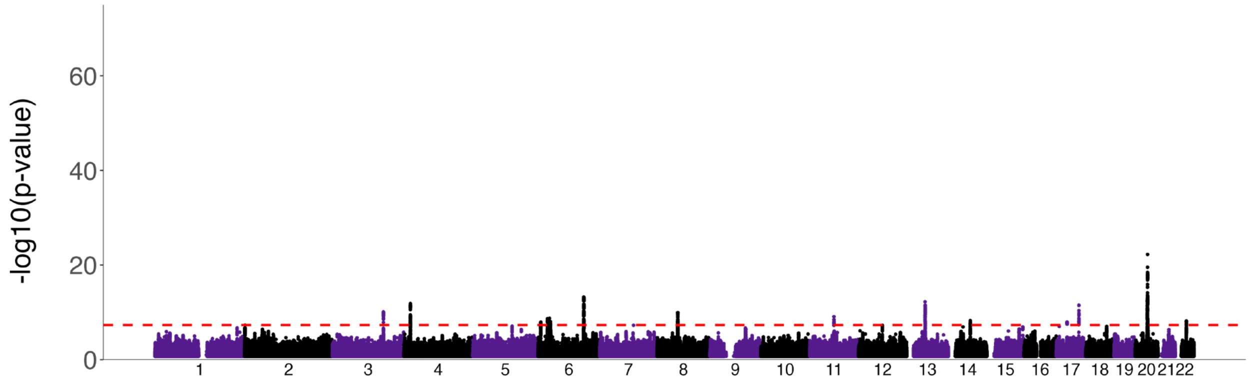

Anterior thigh muscle fat infiltration (%)

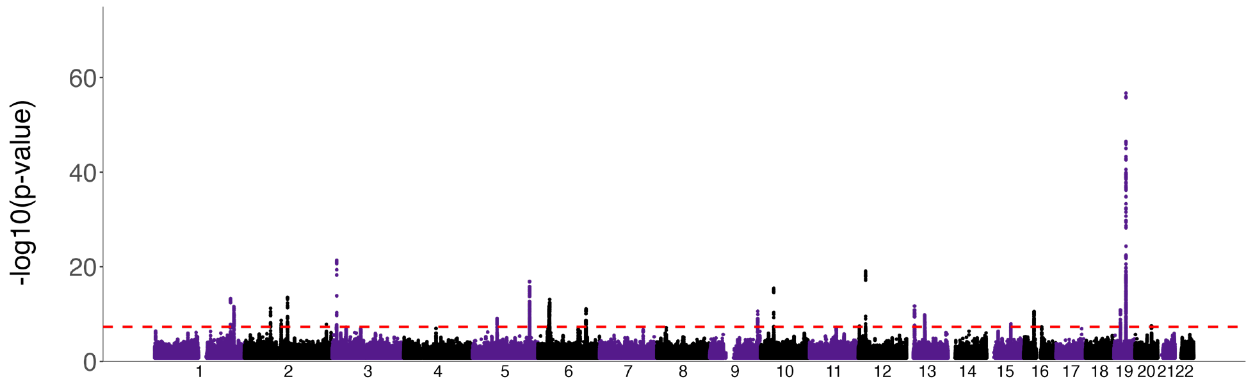

Posterior thigh muscle fat infiltration (%)

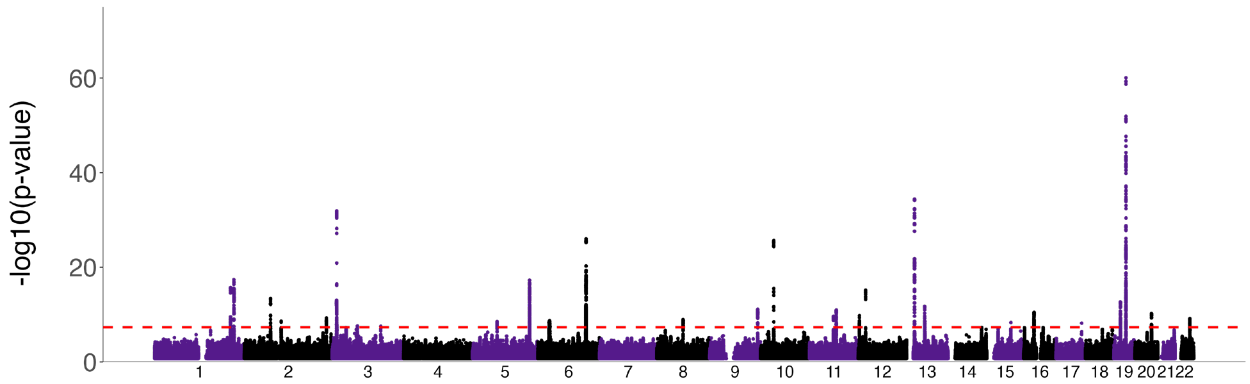

Weight-muscle-ratio

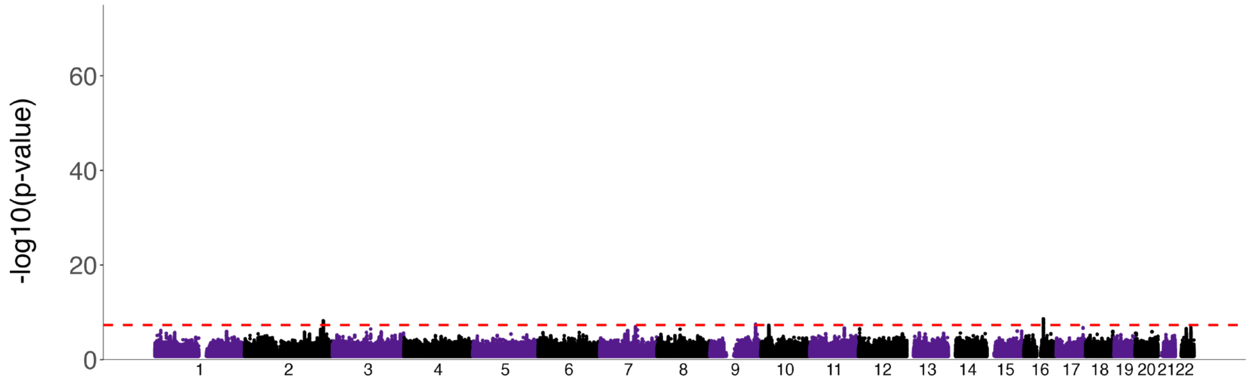

Abdominal fat ratio

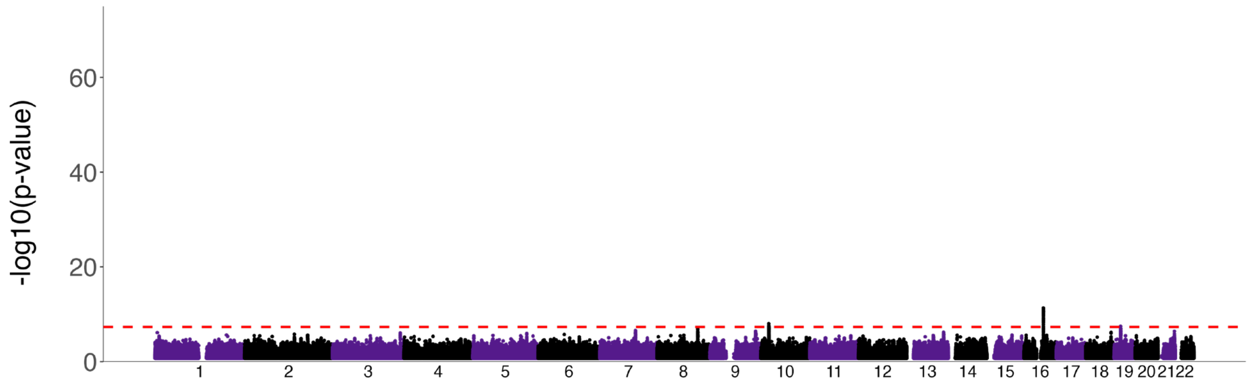

Liver proton density fat fraction (%)

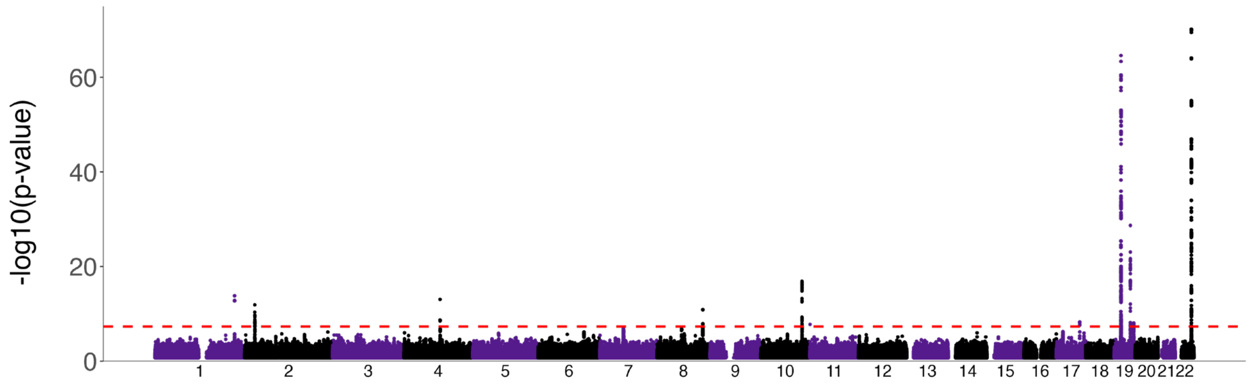

VAT/height^2

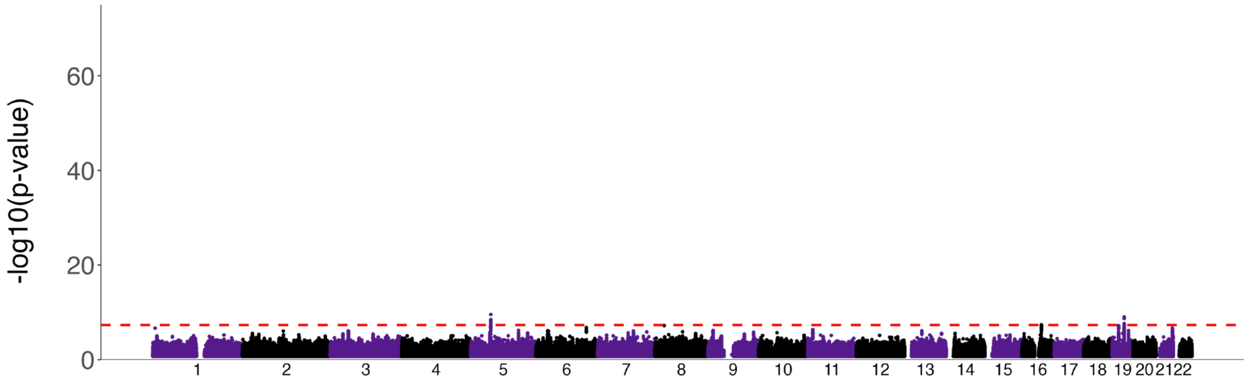

ASAT/height^2

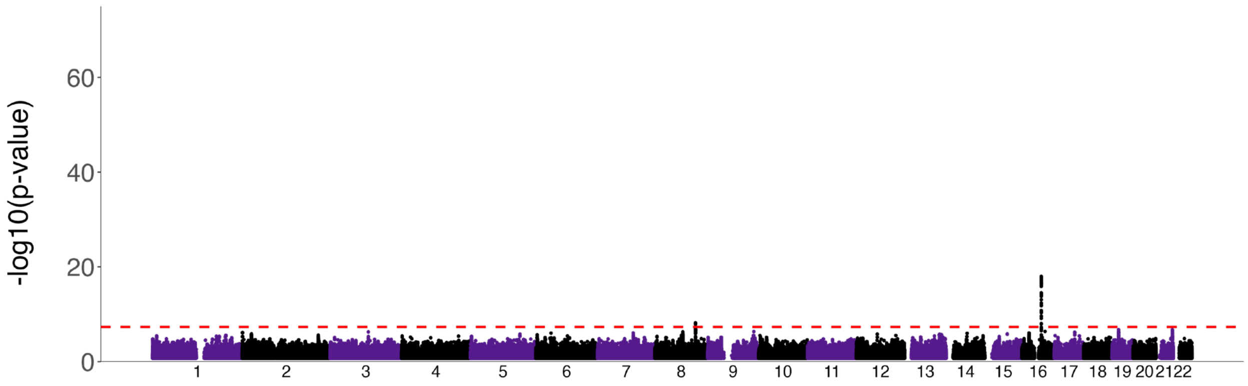

ATMV/height^2

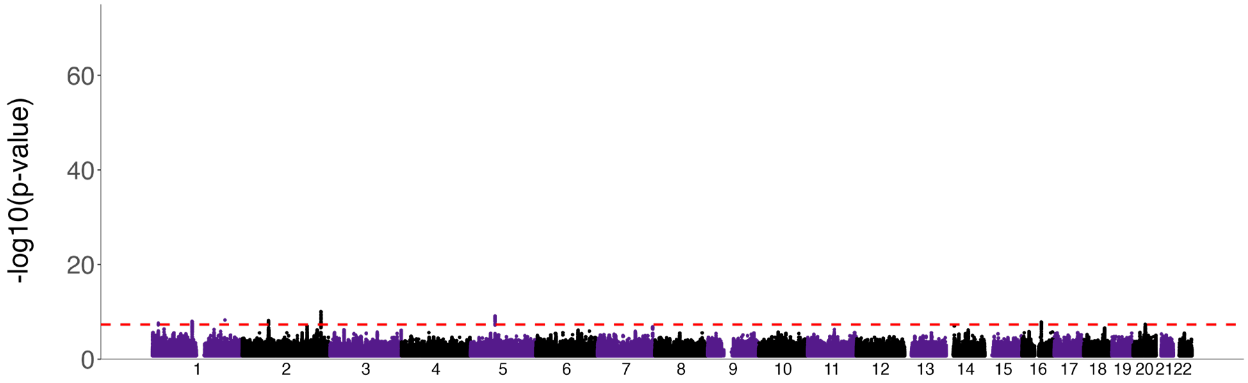

PTMV/height²

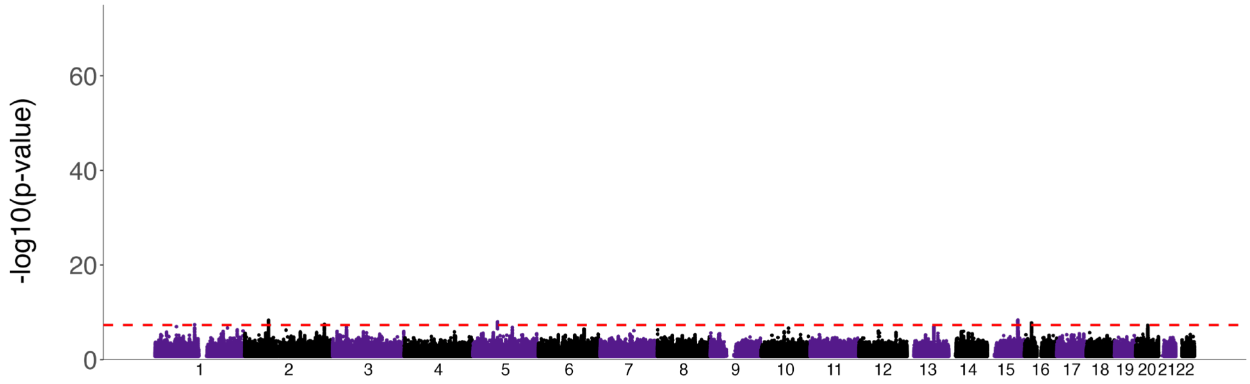

Total thigh muscle volume (TTMVz; age, height, weight and sex corrected)

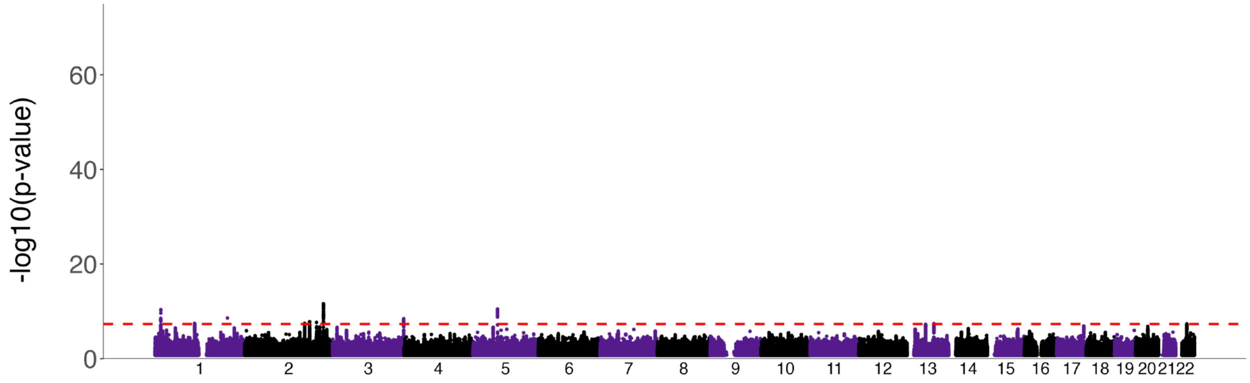

Supplementary Figure 1. Manhattan plots for each of the MRI-derived measures of body composition, as indicated above each of the plots. *The x-axis indicates genomic position, ordered by chromosome, the y-axis indicates the -log10(p). The red horizontal dotted line indicates nominal GWAS significance threshold ($p=5*10^{-8}$).*

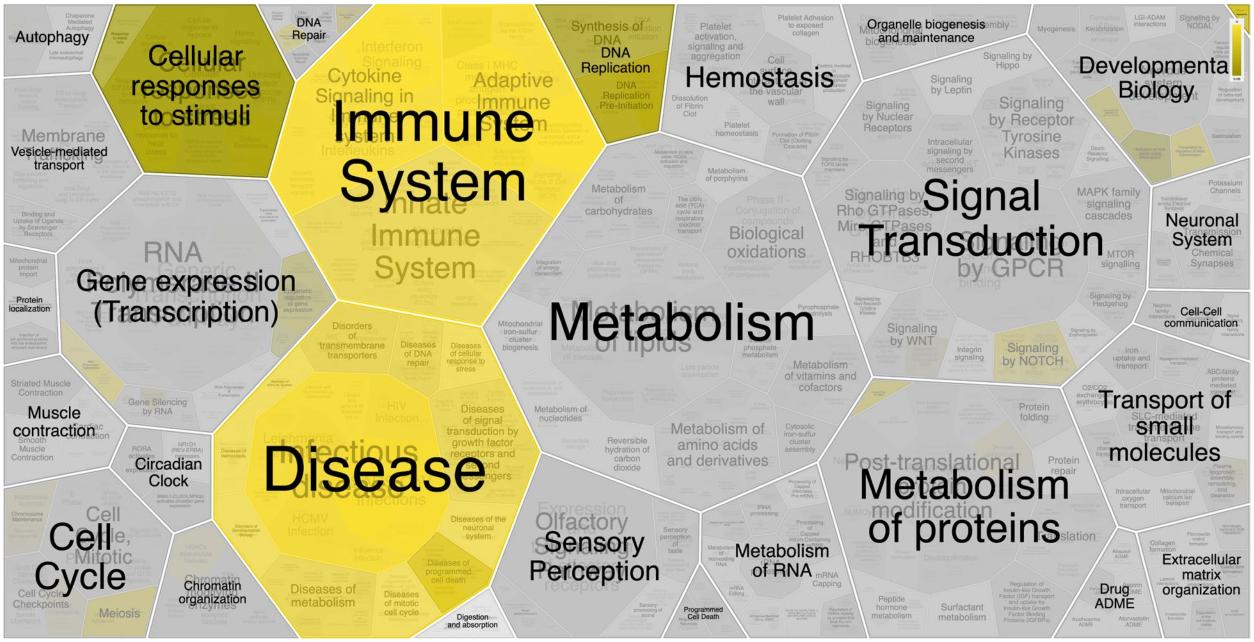

Supplementary Figure 2. Visual representation of the output from coupling the MOSTest-identified set of genes to the Reactome database. *Brighter yellow indicates higher significance. Information on the specific pathways and their significance is provided in the Supplementary Data 5.*

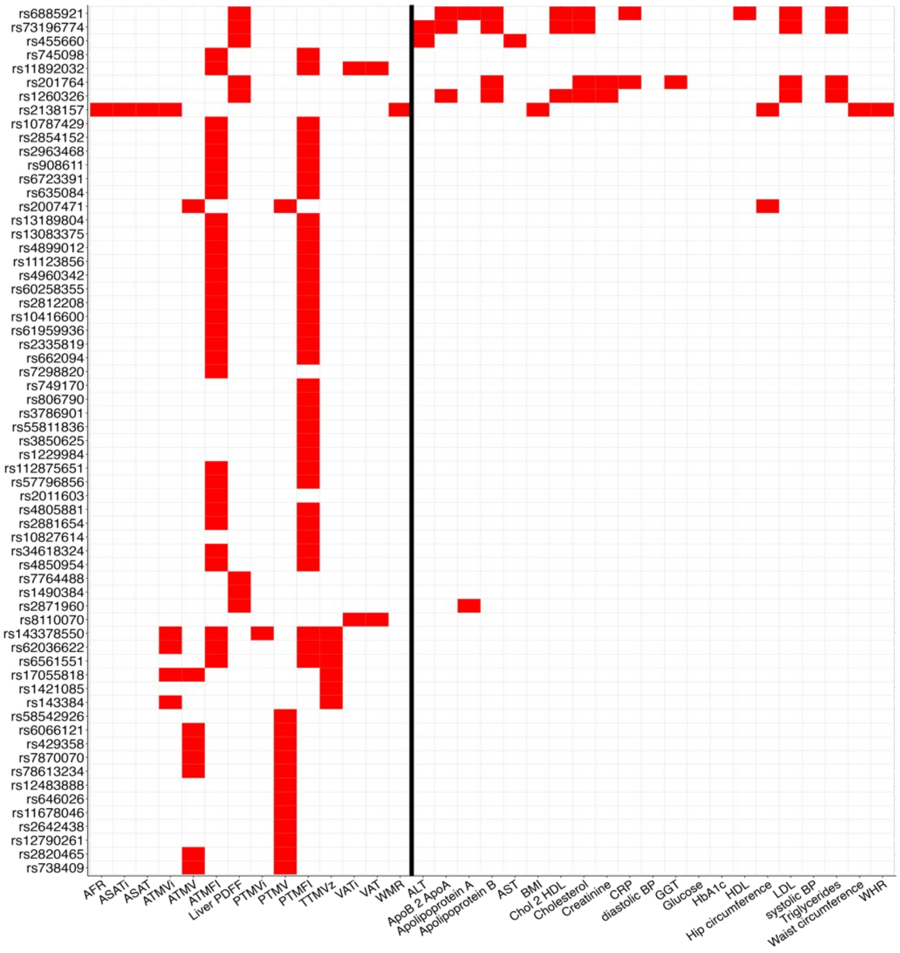

Supplementary Figure 3. Novelty of loci discovered through the univariate GWAS of body MRI measures. *The rs-codes on the y-axis represent the lead SNPs of the discovered loci, and on the x-axis are all the studied measures; on the left of the vertical black line are the body MRI measures and on the right are the secondary measures of cardiometabolism. Red cells indicate that the SNP is whole-genome significantly associated with the measure.*

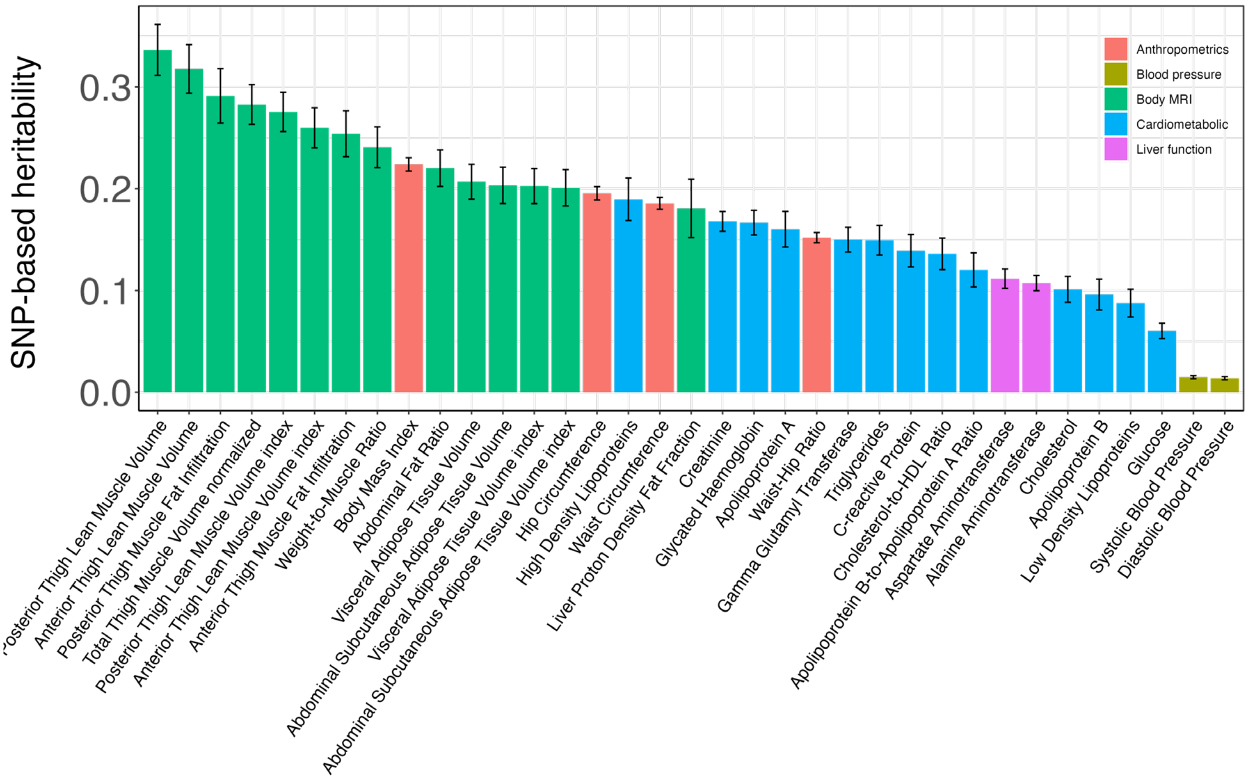

Supplementary Figure 4. Bar plot summarizing the SNP-based heritability (y-axis) for each of the univariate measures analyzed (x-axis), as calculated through LD score regression. *This tool was applied to the GWAS summary statistics, derived from N=33 588 individuals. The fill of the bars indicates the measurement category, as indicated in the legend. The error bars reflect standard error.*

Supplementary Table 1. Results from sex-stratified GWAS on the body MRI measures, listing locus yields and genetic correlations (r_g) between the male and female-specific GWAS.

Measure	r_g	Male yield	Female yield
Abdominal Fat Ratio	0.84	0	0
Abdominal Subcutaneous Adipose Tissue Volume index	0.79	1	1
Abdominal Subcutaneous Adipose Tissue Volume	0.73	1	1
Anterior Thigh Lean Muscle Volume index	0.56	0	0
Anterior Thigh Lean Muscle Volume	0.63	3	1
Anterior Thigh Muscle Fat Infiltration	0.89	6	6
Liver Proton Density Fat Fraction	0.8	6	3
Posterior Thigh Lean Muscle Volume index	0.58	0	0
Posterior Thigh Lean Muscle Volume	0.6	2	3
Posterior Thigh Muscle Fat Infiltration	0.97	7	11
Total Thigh Muscle Volume normalized	0.73	0	0
Visceral Adipose Tissue Volume index	0.76	0	0
Visceral Adipose Tissue Volume	0.78	0	0
Weight-to-Muscle Ratio	0.82	0	0
