# Supplementary material for: The link between liver fat and cardiometabolic diseases is highlighted by genome-wide association study of MRI-derived measures of body composition
Source: Commun Biol. 2022 Nov 19;5:1271. doi: 10.1038/s42003-022-04237-4 (PMC9675774; doi:10.1038/s42003-022-04237-4)
Supplement: Supplementary file 3 — Description of Additional Supplementary Data [file 42003_2022_4237_MOESM3_ESM.docx]

**Description of Additional Supplementary Files**

**File name:** Supplementary Data 1

**Description:** Overview of the individual loci discovered through the univariate GWAS

**File name:** Supplementary Data 2

**Description:** List of enriched pathways per measure

**File name:** Supplementary Data 3

**Description:** Overview of the most significant biological processes associated with each measure

**File name:** Supplementary Data 4

**Description:** Overview of the loci discovered through the multivariate GWAS

**File name:** Supplementary Data 5

**Description:** Tabulated output from coupling the multivariate GWAS-mapped genes to the Reactome database

**File name:** Supplementary Data 6

**Description:** Overview of the loci discovered through the multivariate GWAS on additional measures related to body composition

**File name:** Supplementary Data 7

**Description:** Heritability estimates per measure, as visualized in Supplementary Figure 4.
