# Supplementary material for: The link between liver fat and cardiometabolic diseases is highlighted by genome-wide association study of MRI-derived measures of body composition
Source: Commun Biol. 2022 Nov 19;5:1271. doi: 10.1038/s42003-022-04237-4 (PMC9675774; doi:10.1038/s42003-022-04237-4)
Supplement: Supplementary file 11 — Reporting Summary [file 42003_2022_4237_MOESM11_ESM.pdf]

Corresponding author(s): Dennis van der Meer
Last updated by author(s): Oct 27, 2022

Reporting Summary

Nature Portfolio wishes to improve the reproducibility of the work that we publish. This form provides structure for consistency and transparency in reporting. For further information on Nature Portfolio policies, see our Editorial Policies and the Editorial Policy Checklist.

Statistics

For all statistical analyses, confirm that the following items are present in the figure legend, table legend, main text, or Methods section.

n/a	Confirmed	
☐	☒	The exact sample size (*n*) for each experimental group/condition, given as a discrete number and unit of measurement
☐	☒	A statement on whether measurements were taken from distinct samples or whether the same sample was measured repeatedly
☐	☒	The statistical test(s) used AND whether they are one- or two-sided<br>*Only common tests should be described solely by name; describe more complex techniques in the Methods section.*
☐	☒	A description of all covariates tested
☐	☒	A description of any assumptions or corrections, such as tests of normality and adjustment for multiple comparisons
☐	☒	A full description of the statistical parameters including central tendency (e.g. means) or other basic estimates (e.g. regression coefficient) AND variation (e.g. standard deviation) or associated estimates of uncertainty (e.g. confidence intervals)
☐	☒	For null hypothesis testing, the test statistic (e.g. *F*, *t*, *r*) with confidence intervals, effect sizes, degrees of freedom and *P* value noted<br>*Give P values as exact values whenever suitable.*
☒	☐	For Bayesian analysis, information on the choice of priors and Markov chain Monte Carlo settings
☒	☐	For hierarchical and complex designs, identification of the appropriate level for tests and full reporting of outcomes
☐	☒	Estimates of effect sizes (e.g. Cohen's *d*, Pearson's *r*), indicating how they were calculated

Our web collection on statistics for biologists contains articles on many of the points above.

Software and code

Policy information about availability of computer code

Data collection	This is an analysis of previously collected magnetic resonance imaging and genetics data. Details on data collection are provided in the Online Methods and the references cited therein.
Data analysis	- PLINK 2<br>- MOSTest<br>- FUMA v1.3.5<br>- MsigdB v5.2<br>- Custom scripts in R 4.0, using packages ggplot2<br>- Matlab 2018

For manuscripts utilizing custom algorithms or software that are central to the research but not yet described in published literature, software must be made available to editors and reviewers. We strongly encourage code deposition in a community repository (e.g. GitHub). See the Nature Portfolio guidelines for submitting code & software for further information.

Data

Policy information about availability of data

All manuscripts must include a data availability statement. This statement should provide the following information, where applicable:

- Accession codes, unique identifiers, or web links for publicly available datasets
- A description of any restrictions on data availability
- For clinical datasets or third party data, please ensure that the statement adheres to our policy

The data incorporated in this work were gathered from public resources. The code is available via https://github.com/precimed/mostest (GPLv3 license), and GWAS

summary statistics are uploaded to the GWAS catalog (https://www.ebi.ac.uk/gwas/). Correspondence and requests for materials should be addressed to d.v.d.meer@medisin.uio.no

Field-specific reporting

Please select the one below that is the best fit for your research. If you are not sure, read the appropriate sections before making your selection.

☒ Life sciences ☐ Behavioural & social sciences ☐ Ecological, evolutionary & environmental sciences

For a reference copy of the document with all sections, see nature.com/documents/nr-reporting-summary-flat.pdf

Life sciences study design

All studies must disclose on these points even when the disclosure is negative.

Sample size	No statistical methods were used to pre-determine sample sizes. We included as much data as we could gather, the sample size is thus based on data availability.
Data exclusions	For this study, we selected 33,588 unrelated White Europeans that had undergone the whole-body MRI protocol, and had complete data. In other words, we excluded those with a different ancestry (N=5,042), or with missing processed outcome measures or covariates (N=4,318).
Replication	We replicated in an additional sample of N=5,081 individuals, processed through identical pipelines. We report how many of whole-genome significant SNPs in the discovery sample are also significant in the replication sample.
Randomization	Randomization is not applicable, as there was no assignment to groups
Blinding	Blinding is not applicable, as there was no assignment to groups

Reporting for specific materials, systems and methods

We require information from authors about some types of materials, experimental systems and methods used in many studies. Here, indicate whether each material, system or method listed is relevant to your study. If you are not sure if a list item applies to your research, read the appropriate section before selecting a response.

Materials & experimental systems

n/a	Involved in the study	
☒	☐	Antibodies
☒	☐	Eukaryotic cell lines
☒	☐	Palaeontology and archaeology
☒	☐	Animals and other organisms
☐	☒	Human research participants
☒	☐	Clinical data
☒	☐	Dual use research of concern

Methods

n/a	Involved in the study	
☒	☐	ChIP-seq
☒	☐	Flow cytometry
☐	☒	MRI-based neuroimaging

Human research participants

Policy information about studies involving human research participants

Population characteristics	We selected unrelated White Europeans that had undergone the body MRI protocol, with available genetic and complete covariate data (N=33588, mean age 64.5 years (SD=7.5), 51.4 % female). For the replication analyses, we made use of data from unrelated non-White Europeans (N=5 042, mean age 63.0 years (SD=7.7), 52.9 % female).
Recruitment	The participants were obtained from the UK Biobank, which is a population-based cohort, on a voluntary basis. Recruitment procedures are described extensively in the UK Biobank design paper, referenced in the manuscript. the participants are known to be of somewhat above average health.
Ethics oversight	National Health Service National Research Ethics Service (ref 11/NW/0382)

Note that full information on the approval of the study protocol must also be provided in the manuscript.

Magnetic resonance imaging

Experimental design

Design type	Structural scans

March 2021

Design specifications	N/A
Behavioral performance measures	N/A

Acquisition

Imaging type(s)	Structural
Field strength	1.5 Tesla
Sequence & imaging parameters	body dual-echo Dixon Vibe protocol and a single-slice multi-echo gradient Dixon acquisition
Area of acquisition	Whole-body

Diffusion MRI ☐ Used ☒ Not used

Preprocessing

Preprocessing software	We obtained preprocessed data from AMRA (Linköping, Sweden; https://www.amramedical.com)
Normalization	non-rigid registration of atlases to acquired image volumes
Normalization template	AMRA
Noise and artifact removal	intensity inhomogeneity correction and visual inspection for segmentation accuracy and manual adjustment
Volume censoring	N/A

Statistical modeling & inference

Model type and settings	Multivariate
Effect(s) tested	Effect of each SNP, across the genome

Specify type of analysis: ☐ Whole brain ☒ ROI-based ☐ Both

Anatomical location(s): Definitions according to AMRA publications (https://www.amramedical.com)

Statistic type for inference (See Eklund et al. 2016)	Permutation-based
Correction	Bonferonni correction (p=5*10-8)

Models & analysis

n/a	Involved in the study
☒	☐ Functional and/or effective connectivity
☒	☐ Graph analysis
☐	☒ Multivariate modeling or predictive analysis

Multivariate modeling and predictive analysis

Let z_{ij} be the value of signed test statistic (z-score) calculated from the univariate association test between j-th SNP and i-th phenotype. Let $z_j=(z_{1j},\dots,z_{Kj})$ be the vector of z-scores of j-th SNP across K phenotypes. Let $Z=\{z_{ij}\}$ be the matrix of z-scores, with rows corresponding to SNPs, and columns corresponding to phenotypes. Further, let $\tilde{Z}=\{\tilde{z}_{ij}\}$ be the matrix of z-scores, calculated from association tests on a randomly permuted genotype vector of each SNP. To preserve correlation structure among phenotypes, the permutation was performed only once for each SNP, and the resulting genotype vector was used in association test across all phenotypes.
The MOSTest test statistic, X_j^2, for the j-th SNP is calculated as Mahalanobis norm $X_j^2=z_j^T \tilde{R}^{-1} z_j$, where $\tilde{R}$ is the KxK correlation matrix of $\tilde{Z}$. The null hypothesis of the MOSTest is that z_j is distributed as a multivariate normal random variable with zero mean and covariance $\tilde{R}$. To compute the theoretical (i.e., under null) p-value of the MOSTest test statistic, we calculated the tail probability that a Chi-square statistics exceeds X_j^2. This probability is given by chi-square distribution with N degrees of freedom, or, equivalently, a gamma distribution, Gamma(K/2,0.5)26. Instead of using theoretical values, we fit the two free parameters of the Gamma(a,b) distribution to the observed distribution of X_j^2 under permutation (shown in Table S4). The p-value of the MOSTest test statistic is then obtained from a cumulative distribution function of the gamma distribution, $p_{MOST}=CDF_{gamma(a,b)}(z_j^T \tilde{R}^{-1} z_j)$.
Controlling for covariates, such as genetic principal components, is done via pre-residualization of all phenotype vectors, i.e. we replace them with the corresponding residual after multiple linear regression of the phenotype vector on the covariates. Additionally, we perform a rank-based inverse normal transformation of the residualized phenotypes, to ensure that z-scores forming the input to MOSTest are

normally distributed.
